# High-yielding nitrate transporter cultivars also mitigate methane and nitrous oxide emissions in paddy

**DOI:** 10.3389/fpls.2023.1133643

**Published:** 2023-02-22

**Authors:** Muhammad Faseeh Iqbal, Yong Zhang, Pulin Kong, Yulong Wang, Kaixun Cao, Limei Zhao, Xin Xiao, Xiaorong Fan

**Affiliations:** ^1^ National Key Laboratory of Crop Genetics, Germplasm Enhancement and Utilization, College of Resources and Environmental Sciences, Nanjing Agricultural University, Nanjing, China; ^2^ Key Laboratory of Plant Nutrition and Fertilization in Lower-Middle Reaches of the Yangtze River, Ministry of Agriculture, College of Resources and Environmental Sciences, Nanjing Agricultural University, Nanjing, China; ^3^ Institute of Food Crops, Jiangsu Academy of Agricultural Sciences, Jiangsu High Quality Rice Research and Development Center, Nanjing Branch of China National Center for Rice improvement, Nanjing, China; ^4^ College of Resource and Environment, Anhui Science and Technology University, Chuzhou, China; ^5^ College of Resource and Environment, Anqing Normal University, Anqing, China; ^6^ Zhongshan Biological Breeding Laboratory, Nanjing, China

**Keywords:** sustainable agriculture, food security, greenhouse gases mitigation, climate-smart strategies, methanogens, nitrate transporters

## Abstract

Development of high yield rice varieties is critical to ensuring global food security. However, the emission of greenhouse gases (GHG) such as Methane (CH_4_) and Nitrous oxide (N_2_O) from paddy fields threatens environmental sustainability. In this study, we selected overexpressed high-affinity nitrate transporters (NRT2.3 along with their partner protein NAR2.1) cultivars, which are effective nitrogen use efficient transgenic lines *pOsNAR2.1: OsNAR2.1* (Ox2) and *p35S:OsNRT2.3b* (O8). We used high (270 kg N/ha) and low (90 kg N/ha) nitrogen (N) fertilizers in paddy fields to evaluate morphophysiological traits, including GHG emission. We found that Ox2 and O8 reduced CH_4_ emissions by 40% and 60%, respectively, compared to their wild type (WT). During growth stages, there was no consistent N_2_O discharge pattern between WT and transgenics (Ox2, O8) in low and high N application. However, total cumulative N_2_O in a cropping season reduced in O8 and increased in Ox2 cultivars, compared to WT. Root aerenchyma formation reduced by 30-60% in transgenic lines. Methanogens like *mcrA* in low and high N were also reduced by up to 50% from rhizosphere of Ox2 and O8. However, the nitrifying bacterial population such as *nosZ* reduced in both transgenics significantly, but *nirK* and *nirS* did not show a consistent variation. The high yield of transgenic rice with limited aerenchyma mitigates the discharge of CH_4_ and N_2_O by reducing root exudates that provide substrates for GHG. Our results improve understanding for breeders to serve the purpose of sustainable development.

## Introduction

Greenhouse gases (GHG) pose a significant threat to the environment with nitrous oxide (N_2_O) and methane (CH_4_) are among the chief contributors to global warming. The global warming potential of CH_4_ and N_2_O is 28 and 265 times that of carbon dioxide (CO_2_) over a 100-year period (Liu S. et al., 2016; IPCC, 2014). Rice cultivation is a major contributor to GHG emission, with paddy fields responsible for 48% of CH_4_ and 50% of N_2_O emission among total agricultural emissions (Oo et al., 2018; Iqbal et al., 2021). Rice used as a staple food for almost half of the global population. It projected 60% increase in rice production in next few decades to meet rapidly growing demand (Dong et al., 2011; Chen et al., 2016; Frona et al., 2019). Excessive production could lead to a 60% increase in anthropogenic GHG emission (Hofstra and Vermeulen, 2016; Frona et al., 2019). To address this, research on balancing GHG mitigation and high yield is inevitable for the development of modern agriculture techniques (Oo et al., 2018).

The agriculture environment such as periodic wet/dry conditions, intensive organic material and fertilizers application like nitrogen creates suitable conditions for CH_4_ and N_2_O discharge in paddy conditions (Akiyama et al., 2005; Hu et al., 2012; Kritee et al., 2018). Nitrogen (N) is vital for plant growth and development, as it is an essential element in the amino acids that make up cell structures and proteins. It is also a key component of many chemical and biological process in plants, including the production of chlorophyll, the compound that allows plants to use sunlight to produce sugars from carbon dioxide and water (Xu et al., 2012; Leghari et al., 2016; Xuan et al., 2017). Rice is traditionally grown in flooded conditions (anaerobic soil), where ammonium is the primary source of N. In such conditions, aerenchyma cells in the shoots transfer O_2_ from shoot to root and into rhizosphere, where process of nitrification (ammonium to nitrate) occurs (Shimamura et al., 2002; Li et al., 2008). Nitrification in paddy fields can provide 25-40% of the total N taken up in the form of nitrate, primarily through a high-affinity transport system (HATS). Additionally, NUE lines facilitate uptake of phosphorus, potassium and other micro nutrients (Fan et al., 2016). These lines not only contribute to plant and root development but also produce green cultivars with less greenhouse effects. Therefore, the benefits of using nitrogen efficient cultivars extend beyond facilitating O_2_ transport and N uptake (Hirel et al., 2007; Leghari et al., 2016; Naz et al., 2019; Zhang et al., 2021).

Elevated CO_2_, N and microbial biomass in rhizosphere are vital for soil GHG emission. Therefore, addressing these areas to mitigate GHG emission is a useful approach (Yang et al., 2022). Several effective methods have been reported to reduce GHG emission, such as management practices, effective drainage, controlling soil microbial and chemical properties, roots aerenchyma, introducing genetically modified crops to reduce usage of fertilizers (Thomson et al., 2012; Carlson et al., 2016; Lévesque et al., 2020; Iqbal et al., 2021). Aerenchyma in plants not only removes gases (such as N_2_O, CH_4_, ethylene, CO_2_, and H_2_O_2_) into the rhizosphere, but also provides a channel for gaseous exchange between the aerial and flooded parts in submerged plants, like rice. Furthermore, it facilitates oxygen diffusion to the root tip (Armstrong, 1980; Justin and Armstrong, 1991). Microbes play an essential role in fixing GHG in soil during paddy conditions. In waterlogged conditions, N_2_O discharge is possible after microbial transformation of N in the soil and nutrients. It is often increased where N availability surpasses plant requirements like in paddy conditions (Smith et al., 2008). In case of CH_4_, organic materials decomposed during anaerobic conditions and CH_4_ gas is produced after oxidation (Smith et al., 2008). The N_2_O emission from agriculture is primarily owed to ammonia oxidation and bacterial fixation, the initiation to nitrification (Thomson et al., 2012). Methanotrophs are also known as methane-oxidizing bacteria (MOB) use CH_4_ as an energy source and ingest 30% methane before reaching into the atmosphere (Liu et al., 2011; Shrestha et al., 2010; Yang et al., 2014).

A critical soil procedure that is directly affected by changes in plant biotic and abiotic stress is CH_4_ oxidation. A plant stress response is an essential factor for the oxidation of CH_4_ (Zhou et al., 2013). The practice of using high-yielding cultivars (less stress) of rice in agriculture accounts for almost 50% of the recent yield growth in developing countries. These new cultivars mainly focused on increasing the harvest index, but this strategy may also be beneficial for reducing GHG emissions. As harvest index increases with higher plant biomass, it can alternatively decrease the production of root exudates that fuel CH_4_ production (Denier et al., 2002; Su et al., 2015; Jiang et al., 2017). The Key source for N_2_O in the soil is N and the balanced use of N is imperative for mitigating N_2_O emission in agriculture (Thomson et al., 2012). Therefore, in this study, we have used *pOsNAR2.1: OsNAR2.1* (Ox2) (Chen et al., 2017) and *p35S:OsNRT2.3b* (O8) overexpression materials (Fan et al., 2016) previously reported as high-yield nitrate transporter cultivars and very efficient for NUE.

The Hypothesis of this study is that the use of high-yielding N efficient rice cultivars will result in reduced GHG emissions under low and high N regimes through limited aerenchyma formation, methanotrophs population, and efficient utilization of available N in soil. The objectives of this study are:

- To measure the CH_4_, N_2_O, and CO_2_ emission from high-yielding nitrate transporter rice cultivars under low and high N regimes.
- To investigate the effect of high-yielding nitrate transporter rice cultivars on aerenchyma formation in roots and methanotrophs population in rhizosphere.
- To suggest high-yielding cultivars which can also mitigate GHG emission under low and high N regimes, thus contributing to a sustainable environment.

## Materials and methods

### Experimental site and transgenic materials

We selected two different types of transgenic cultivars. The first one was the *OsNAR2.1* overexpression line (Ox2) with a background of *Oryza sativa* L. ssp. *Japonica* cv. Wuyunjing7 as described in (Chen et al., 2017). The second was the *OsNRT2.3b* overexpression plant (O8) with a background of *Oryza sativa* L. ssp. *Japonica* cv. Nipponbare as described in (Fan et al., 2016). Both cultivars (Ox2, O8) have different phenotypic backgrounds; therefore, we used and compared them with their own wildtypes. This experiment was performed in the experimental site of Anhui Science and Technology University in 2017 and 2018. The site is located in Fengyang County, Anhui Province, on south bank of the middle reaches of the Huaihe River, north latitude (32°37′-33°03′N, 117°19′-117°57′E). We also calculated soil chemical properties, the soil was yellow-brown in color, and initial soil profile was as follows: pH 7.54, available phosphorus 393.98mg/kg, available potassium 114.81mg/kg, total nitrogen 0.37g/kg, and organic matter content 8.02g/kg. Soil and plants samples were collected for further experiments in lab and examination of chemical properties before and after rice harvest. The data collection on plant phenotype and agronomic traits in field conditions were recorded following standard protocol (Chen et al., 2016).

### Field experiment

The Plants were planted in a field plots that was fertilized at a rate of 270 kg N/ha for high nitrogen (HN) field and 90kg N/ha for low nitrogen (LN) field. Three plots were used to set the replicates for the test and forty-five seedlings were grown in each plot. the plot area was 3.75 square meters. Before transplantation, nylon bags with a diameter of 30um, a width of 7.5cm, and a height of 12cm were buried around the plot in advance. Each bag contained 475 grams of soil for collecting soil samples during the experiment for GHG emission, aerenchyma formation, and microbial population abundance (Nie et al., 2015; Iqbal et al., 2021). The seedlings were raised in the nursery on May 10-12, transplanted on June 7-9 and harvested on October 20-22 during planting year of 2017 and 2018. A total of 60kg/ha of urea was applied in each plot, 50% was used as a base fertilizer on June 13-14, 20% as a tiller fertilizer on July 1-2, and 30% as a spike fertilizer on August 28-29. During the basal fertilizer period, 75 kg/ha of superphosphate (containing 12% P_2_O_5_) and 150kg/ha of potassium chloride (containing 60% K_2_O) were applied at one time. Field management and farming practices were followed according to local agricultural practices.

### Methane, carbon dioxide and nitrous oxide flux measurements

The simultaneous determination of methane, carbon dioxide and nitrous oxide fluxes was calculated by using static black chamber method. We used closed box-gas chromatography (Zhang et al., 2015). Before transplanting rice to the plot, a PVC flux loop was permanently embedded in each plot to continuously monitor GHG emissions during the experiment period. A 5cm deep groove on the edge of each base was used to inject water and seal the gas chamber during gas production, in order to prevent gas exchange. The cross-sectional area of the gas tank was 0.25m^2^ (50cm*50cm) and the height was 50cm. Once the rice grew taller, the height was increased to 1m.

Sponge and aluminum foil were wrapped around the exterior of the gas box to prevent drastic changes in temperature inside the chamber. The gas chamber was also equipped with a small fan to ensure that the gas in the box was fully mixed. Sampling was conducted 9:00 a.m. and 11:00 a.m. every time, using a 60ml medical syringe to collect gas from the top of the chamber, once every 5 minutes, for a total of four times (Hao et al., 2001).

Before gas chromatography analysis, the gas was transferred to a vacuum airbag for less than a day to ensure that the gas was not mixed with the outside environment. A gas chromatograph was equipped with an electron capture detector (ECD) and a flame ionization detector (Agilent 7890A network gas chromatograph, Gow Mac instruments, Bethlehem, PA, USA). It was used for the simultaneous analysis of methane, carbon dioxide, and nitrous oxide gas concentrations (Liu S. et al., 2016). The linear regression slope for the greenhouse gas concentration of the continuous samples was calculated, and the data with the linear regression value r^2^<0.9 was removed from the dataset for gas flux calculation (Liu et al., 2013). The gas flux calculation formula is as follows:


F=H×ρ×(273.2+273.2+T)×(dC/dt)

Where F is the gas emission flux (mg m^-2^ h^-1^), H is the height of the sampling chamber, ρ is the gas density in the standard state, and dC/dt is the slope of the concentration growth of the gas concentration fitted by a linear equation (mg m^-3^ h^-1^). T is the temperature in the sampling chamber at the time of sampling(°C). During the test, cumulative methane, carbon dioxide, and nitrous oxide emissions were sequentially accumulated from the flux of each two adjacent intervals. For N_2_O cumulative emission, the final value was multiplied by atomic mass of N_2_ divided by atomic mass of N_2_O (Liu et al., 2013; Liu S. et al., 2016; Iqbal et al., 2021). The gas flux calculation formula and the total cumulative GHG emission during one growing season was also calculated according to protocol given in (Iqbal et al., 2021).

### Determination and quantification of root aerenchyma

Roots of WT and overexpress lines (5-6cm in length) at the tillering stage (July 9) at the experimental site of Anhui Science and Technology University were collected for the determination of aerenchyma. On the sampling day we selected five to six random plants, WT and overexpress plants’ complete root tips of about 5-6cm in length and the same size were gently removed with a sharp blade and quickly placed into FAA fixative. Before embedding the root slices in resin, the root was divided into six short segments with a length of 0.5cm from the root tip, and the root samples were vacuumed in a vacuum chamber. Then, the root samples were embedded in EPON812 embedding resin (SPI, USA) (Zhu et al., 2015). The resin-embedded root tissues were sectioned using a Leica automated microtome (RM 2265, Leica, Germany), mounted on a microscope slide and dried at 42°C. The cross-sections of root tissues were observed with a fluorescence microscope (OLYMPUS BX51), and the images were recorded from the microscope using the cellSens standard software. Ventilation tissues formation was calculated from section images using Image J^®^ software (Zhu et al., 2015).

### Collection of soil and extraction of its DNA and qPCR analysis

We used WT and transgenic lines to understand microbial population dynamics under field conditions in root zones. We collected soil from the rhizosphere after transplanting seedlings from nursery (day1), at the vegetative stage (day 14 and 28) and at the reproductive stage (day 43). A quantitative analysis of soil microorganism was conducted. We quantified the relative abundance of particulate methane monooxygenase (*pmoA*), methyl coenzyme M reductase (*mcrA*) used as functional marker genes to determine methanotrophs in soil. Nitrite reductase genes (*nirK, nirS*) were used as functional marker genes to determine denitrifying bacteria in soil samples, and for active and total N_2_O consuming bacteria in soil, nitrous oxide reductase genes (*nosZ*) were used as a biomarker. Soil samples were also taken before and after plantation for chemical analysis to determine the nutrient content. Nylon bags were used to collect rhizosphere soil in the field. Fresh soil with a weight of about 0.5g attached to the roots was scratched, and the total DNA was extracted from the soil using FastDNA Spin Kit for soil (MP Biomedicals LLC USA). A QuantStudio 6Flex instrument was used to conduct a soil DNA quantitative experiment, which was repeated twice for each sample. The 20ul qRT-PCR system is as follows: 10.0ul SYBR PremixExTaq (TaKaRa Norrie Biotech Auckland New Zealand), 0.4ul each of the forward and reverse primers, 2.0ul template DNA, 0.4ul DyII, 6.8ul ddH_2_O (Iqbal et al., 2021).

### Statistical analysis

Data were analyzed by using a *t-*test for comparing two groups and one-way analysis of variance (ANOVA) for comparing more than two groups were used followed by Tukey’s test (P<0.05). Significant difference between two groups was shown by small alphabet letters (a,b) like ‘a’ shows higher value and ‘b’ shows smaller value. The data from Ox2 always compared with WT (Wuyunjing7 background) and O8 always compared with WT-N (Nipponbare background). Statistical analyses and Pearson correlation were conducted using the SPSS software (version 25.0) (SPSS Inc., Chicago, USA).

## Results

### Nitrate transporter cultivars (Ox2, O8) reduced greenhouse gases emission in paddy

**Figure 1:**
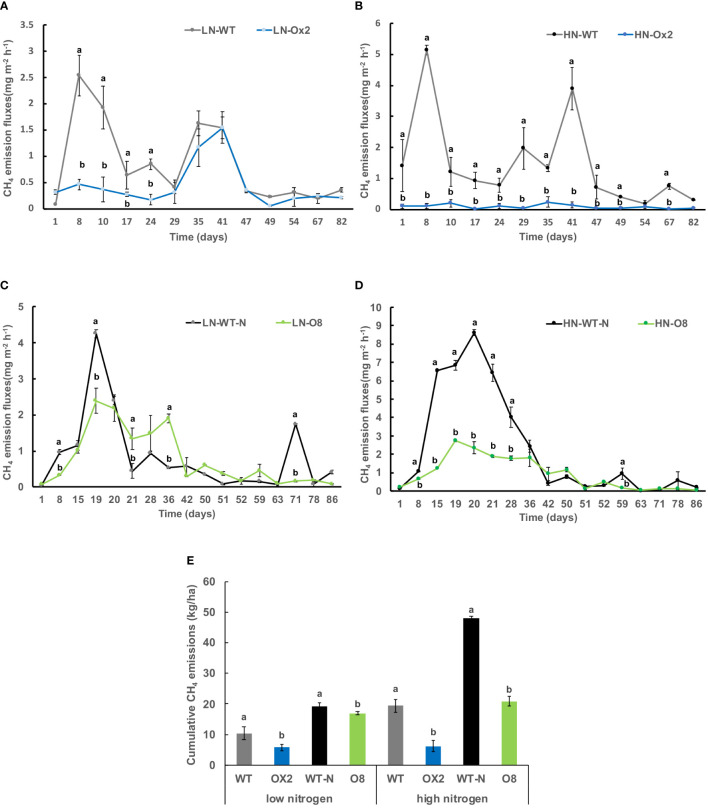
Detailed CH_4_ gas emission fluxes (mgm^-2^h^-1^) from rhizosphere of WT, Ox2, WT-N and O8. The (LN) low nitrogen soil application shows in figure **(A, C)** and (HN) high nitrogen soil application shows in figure **(B, D)**. X-axis shows CH_4_ gas emission recording time (days), from the first measurement after transplanting seedlings from nursery to field is day1 to onwards **(A-D)**. CH_4_ emission flux (mgm^-2^h^-1^) from the rhizosphere was compared between Ox2 in figures a-b and O8 in figure **(C, D)** with their respective wildtypes (WT) separately. Figure **(E)** shows cumulative CH_4_ emission during full growing season in kilogram per hectare (kg/ha). Cumulative emission data consist of 13 individual values taken during various growth stages (n=13). Error bars: SE (n = 3). Significant difference between two cultivars (transgenic and respective WT) are indicated by different letters: a= high value, b= lower value (*t*-test, *p<* 0.05).

We grew plants *pOsNAR2.1:OsNAR2.1* (Ox2) and *p35:OsNRT2.3b* (O8) along with their respective WT to quantify the discharge of GHG emission after planting in paddy field conditions. The details of research plot area and plant material is given in material method section. The first measurement of greenhouse gases was set to day-one after the transplanting seedlings from the nursery to the field. We separately used two kinds of Nitrogen (N) applications low nitrogen (LN) and high nitrogen (HN) in the paddy fields. Whether under high or low N conditions, methane (CH_4_) emission fluxes were lower in overexpression (OE) lines as compared to their WT (**Figure 1**). The CH_4_ discharge in Ox2 was lower than WT at the initial stages of growth, especially during the vegetative stage. However, with the passage of growth, the difference of discharge between Ox2 and its WT was minimal in high and low N conditions (**Figures 1A, B**). Likewise, in the O8 line, CH_4_ emission fluxes show a similar pattern during the vegetative growth. However, in high N conditions, the difference between WT and N increases to more than two folds (**Figures 1C, D**). Total CH_4_ emission significantly increased to two folds in high N conditions in WT compared to OE lines. However, the significant difference of cumulative methane emission in low N application in both Ox2 and O8 compared to their WT was less than 1.5 folds (**Figure 1E**).

**Figure 2:**
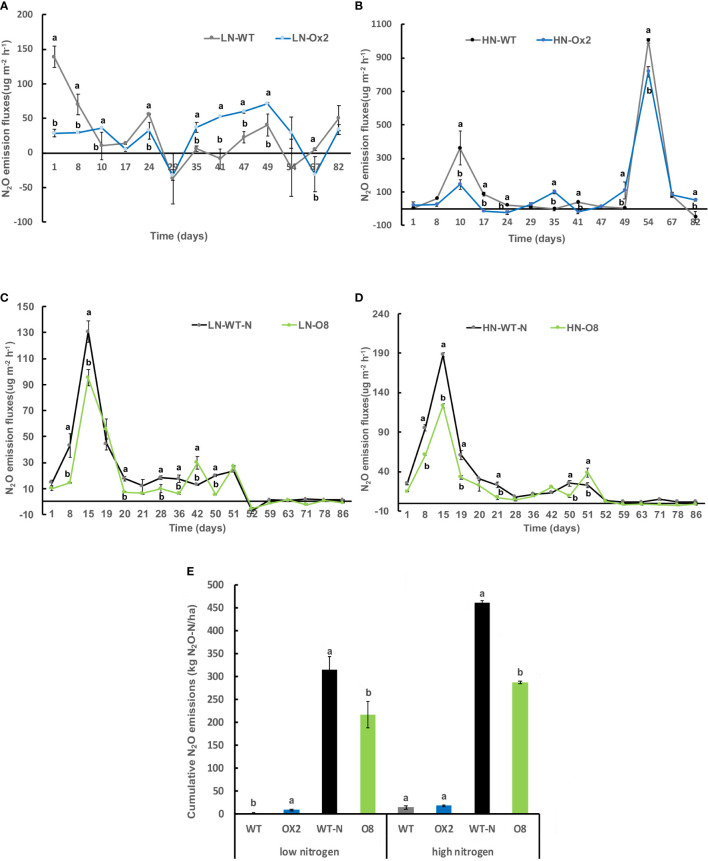
Detailed N_2_O gas emission fluxes (ugm^-2^h^-1^) from rhizosphere of WT, Ox2, WT-N and O8. The (LN) low nitrogen soil application shows in figure **(A, C)** and (HN) high nitrogen soil application shows in figure **(B, D)**. X-axis shows N_2_O gas emission recording time (days), from the first measurement after transplanting seedlings from nursery to field is day1 to onwards **(A-D)**. N_2_O emission flux (ugm^-2^h^-1^) from the rhizosphere was compared between Ox2 in figures **(A, B)** and O8 in figure **(C, D)** with their respective wildtypes (WT) separately. Figure **(E)** shows cumulative N_2_O emission during full growing season (kg N_2_O-N/ha). Cumulative emission data consist of 13 individual values taken during various growth stages (n=13). Error bars: SE (n = 3 Significant difference between two cultivars (transgenic and respective WT) are indicated by different letters: a= high value, b= lower value (*t*-test, *p<* 0.05).

**Figure 3:**
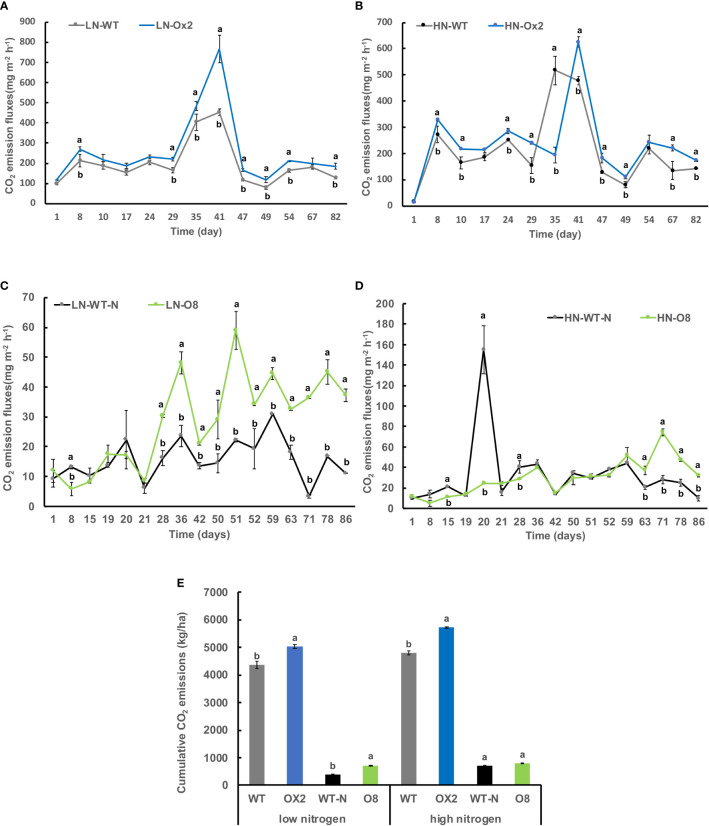
Detailed CO_2_ gas emission fluxes (mgm^-2^h^-1^) from rhizosphere of WT, Ox2, WT-N and O8. The (LN) low nitrogen soil application shows in figure **(A, C)** and (HN) high nitrogen soil application shows in figure **(B, D)**. X-axis shows CO_2_ gas emission recording time (days), from the first measurement after transplanting seedlings from nursery to field is day1 to onwards **(A-D)**. CO_2_ emission flux (mgm^-2^h^-1^) from the rhizosphere was compared between Ox2 in figures **(A, B)** and O8 in figure **(C, D)** with their respective wildtypes (WT) separately. Figure **(E)** shows cumulative CO_2_ emission during full growing season in kilogram per hectare (kg/ha). Cumulative emission data consist of 13 individual values taken during various growth stages (n=13). Error bars: SE (n = 3). Significant difference between two cultivars (transgenic and respective WT) are indicated by different letters: a= high value, b= lower value (*t*-test, *p<* 0.05).

We further calculated nitrous oxide (N_2_O) emission rates among OE lines in high and low N applications. In Ox2 the emission fluxes in N_2_O were significantly lower in HN regime, but it was not consistent during all plant growth stages as compared to WT (**Figures 2A, B**). The N_2_O emission was increased significantly at LN application in Ox2 after 29 days (**Figure 2A**). In contrast, the O8 line shows a significant difference in N_2_O emission rate during different growth stages of paddy (**Figures 2C, D**). The cumulative N_2_O emission rate shows a similar pattern in low and high N applications. The Ox2 line slightly up-regulated the N_2_O emission rate compared to its WT at LN, but the O8 reduced the N_2_O emission significantly in LN and HN emission in its rhizosphere (**Figure 2E**). We also calculated CO_2_ emission fluxes (**Figure 3**). The CO_2_ emission was higher in both transgenic lines (Ox2, O8) as compared to their WT (**Figure 3**). In Ox2 line produced higher amount of CO_2_ as compared to WT both in high and low N regimes (**Figures 3A, B**). Therefore, the cumulative emission from Ox2 was significantly higher as compared to WT in both low and high nitrogen (**Figure 3E**). Similarly, for line O8 significant emission was noticed for CO_2_ emission compared to its WT in low N application but there was not any consistent pattern at high nitrogen application (**Figures 3C, D**). However, the O8 line compared to its WT for cumulative emission was significantly higher in O8 in LN and no difference was recorded for HN (**Figure 3E**).

### Limited aerenchyma formation in Ox2 and O8 cultivars

**Figure 4:**
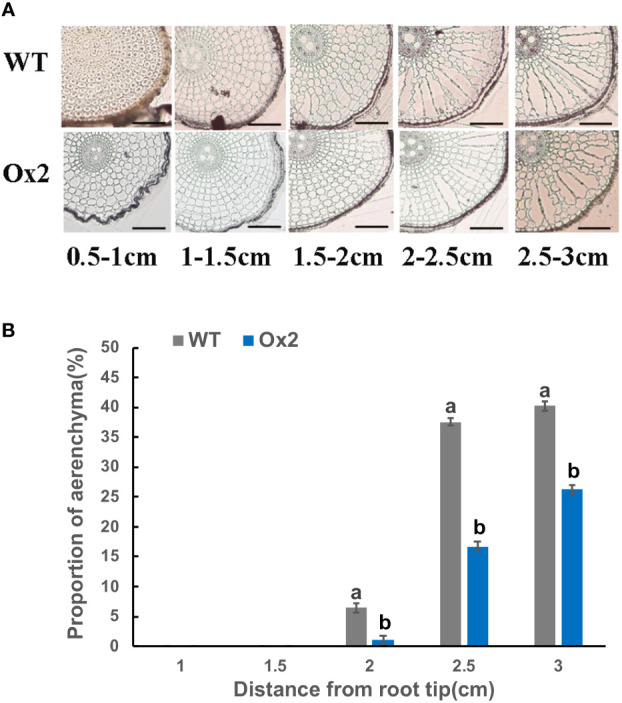
Describe aerenchyma formation in rice roots of WT and Ox2. Figure a-b show resin sections results of roots and aerenchyma formation (%) 0-0.5 cm, 0.5-1.0 cm, 1.0–1.5 cm, 1.5–2.0 cm and 2.0-2.5 cm from root apex in WT and Ox2 and significant difference between WT and Ox2 in each group are indicated by different letters: a= high value, b= lower value. Aerenchyma pics scale bar: 200um, Error bars: SE (n = 3) (*t*-test, *p<* 0.05).

**Figure 5:**
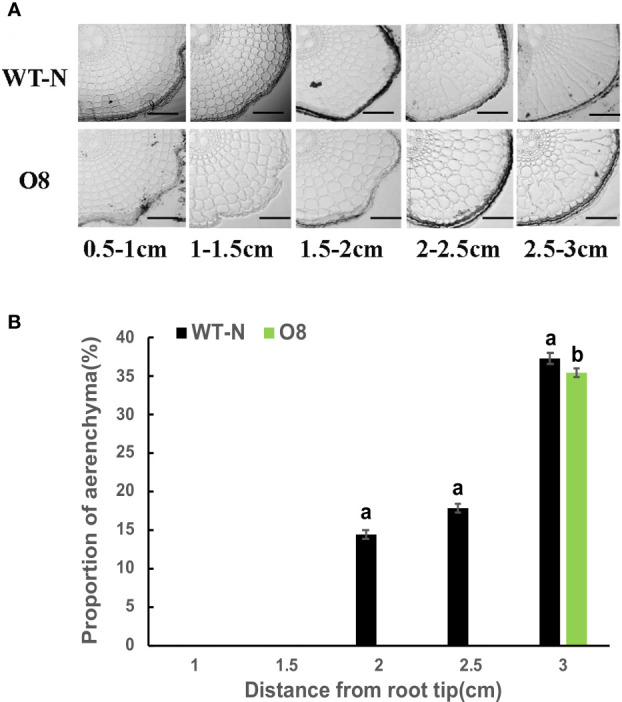
Describe aerenchyma formation in rice roots of WT and O8. Figures **(A, B)** show resin sections results of roots and aerenchyma formation (%) 0-0.5 cm, 0.5-1.0 cm, 1.0–1.5 cm, 1.5–2.0 cm and 2.0-2.5 cm from root apex in WT and O8 and significant difference between WT and O8 in each group are indicated by different letters: a= high value, b= lower value. Aerenchyma pics scale bar: 200um, Error bars: SE (n = 3) (*t*-test, *p<* 0.05).

Root aerenchyma is the passage for the discharge of gases between aerial and flooded parts in a paddy field. Therefore, we observed the development of aerenchyma among Ox2 and O8 lines. The rice root system develops during the tillering stage; we collected rice root samples to observe the aerenchyma. Through root sections, we can see that aerenchyma of WT developed earlier than Ox2 (**Figure 4**). At the distance of 0.5-1.0cm from the root tip, aerenchyma of WT began to form while Ox2 was not formed yet. Root aerenchyma formation was increased with continuous root development. The aerenchyma formation percentage was significantly higher in WT than Ox2, even at different distances from the root apex (**Figures 4A, B**). The aerenchyma formation in WT at the distance of 1.5-2.5 cm from the root apex was twofold increased as compared to Ox2 (**Figures 4A,B**). During the aerenchyma study in O8 line, we observed a similar pattern (**Figure 5**). At the distance of 0.5-2.0 cm from the apex, the aerenchyma formation was significantly lower in O8 lines than WT (**Figures 5A,B**). At the distance of 2.0 to 3.0 cm from the root tip, the aerenchyma formation showed a similar downward percentage in the O8 line than WT (**Figures 5A,B**). Therefore, it can speculate that transgenic rice with overexpression of *OsNAR2.1* and *OsNRT2.3b* regulates the formation of aerenchyma structure somehow. This may be regulated directly or through interaction with related genes. As for the specific molecular mechanism, it requires further investigation.

### Nitrate transporter cultivars (Ox2, O8) regulated methanotrophs and nitrifying bacterial population in soil

**Figure 6:**
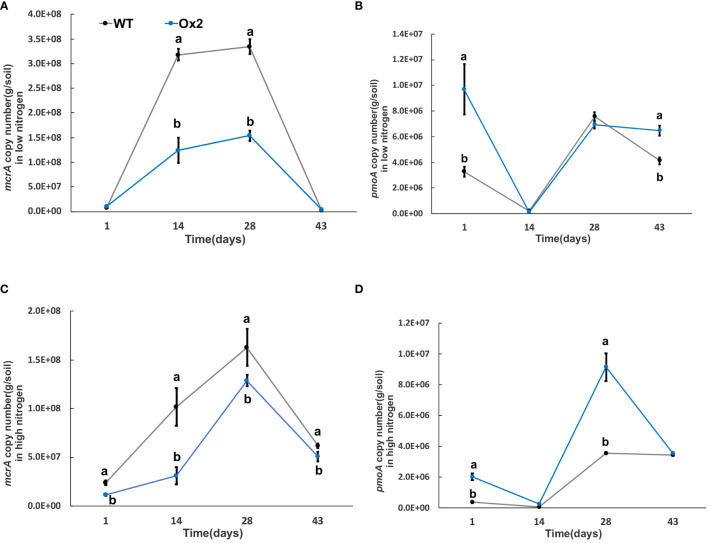
The abundance of *mcrA and pmoA* genes in low and high Nitrogen soil of WT and Ox2. X-axis shows the soil sampling time (days), from the first measurement after transplanting seedlings from nursery to field. Figures **(A, C)** show the *mcrA* copy number/g of soil. Figures **(B, D)** show the *pmoA* copy number/g of soil. Error bars: SE (n = 3). Significant difference between WT and Ox2 are indicated by different letters: a= high value, b= lower value (*t*-test, *p<* 0.05).

**Figure 7:**
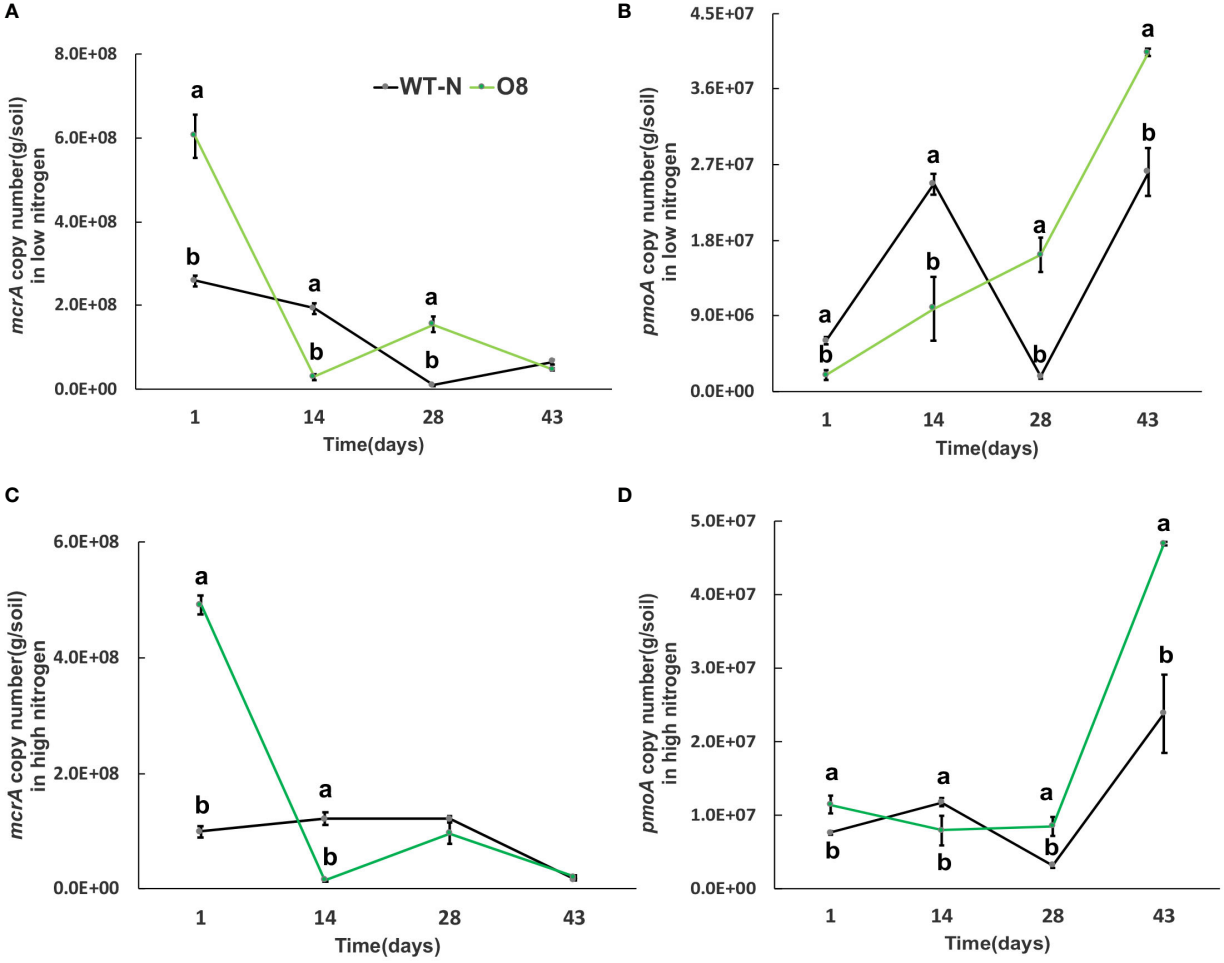
The abundance of *mcrA and pmoA* genes in low and high Nitrogen soil of WT-N and O8. X-axis shows the soil sampling time (days), from the first measurement after transplanting seedlings from nursery to field. Figures **(A, C)** show the *mcrA* copy number/g of soil. Figures **(B, D)** show the *pmoA* copy number/g of soil. Error bars: SE (n = 3). Significant difference between WT and O8 are indicated by different letters: a= high value, b= lower value (*t*-test, *p<* 0.05).

**Figure 8:**
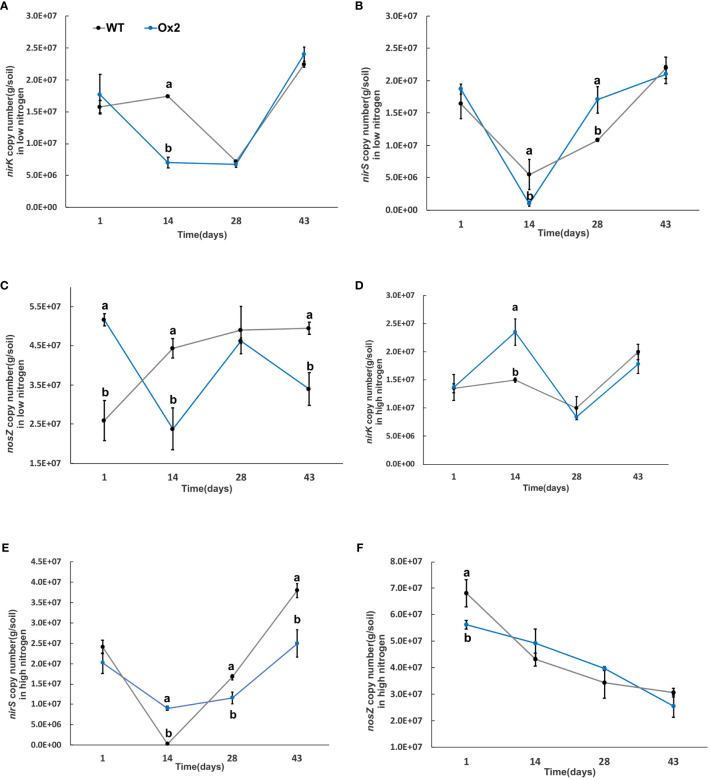
The abundance of *nirK, nirS and nosZ* genes in low and high Nitrogen soil of WT and Ox2. X-axis shows the soil sampling time (days), from the first measurement after transplanting seedlings from nursery to field, Figures **(A, D)** show the *nirK* copy number/g of soil in low and high nitrogen application. Figures **(B, E)** show the *nirS* copy number/g of soil in low and high nitrogen application. Figures **(C, F)** show the *nosZ* copy number/g of soil in low and high nitrogen application Error bars: SE (n = 3). Significant difference between WT and Ox2 are indicated by different letters: a= high value, b= lower value (*t*-test, *p<* 0.05).

**Figure 9:**
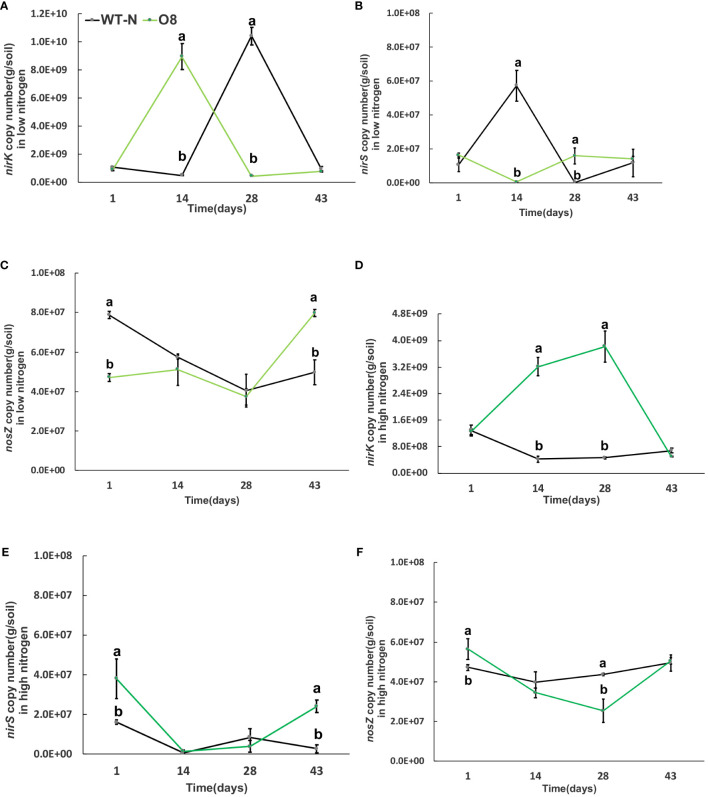
The abundance of *nirK, nirS and nosZ* genes in low and high Nitrogen soil of WT-N and O8. X-axis shows the soil sampling time (days), from the first measurement after transplanting seedlings from nursery to field. Figures **(A, D)** show the *nirK* copy number/g of soil in low and high nitrogen application. Figures **(B, E)** show the *nirS* copy number/g of soil in low and high nitrogen application. Figures **(C, F)** show the *nosZ* copy number/g of soil in low and high nitrogen application Error bars: SE (n = 3). Significant difference between WT and O8 are indicated by different letters: a= high value, b= lower value (*t*-test, *p<* 0.05).

Greenhouse gas emissions from rice fields are also associated with multiple microorganisms; methanogenic bacteria *mcrA* and *pmoA* are important methanotrophs. In addition, nitrification and denitrification bacteria; *nirK*, *nirS* and *nosZ* are involved in the generation and oxidation of greenhouse gases such as N_2_O. The significant difference was calculated in transgenic lines after transplanting from nursery to field called day 1 (**Figures 6**–**9**). The abundance of *mcrA* copy number was significantly decreased in Ox2 both in low and high N regimes (**Figures 6A, C**). The difference was maximum during the vegetative growth stage. However, in the O8 line, the *mcrA* copy number was not consistent even in high or low N applications (**Figures 7A,C**). The abundance of the *pmoA* microbial population regulated significantly even in high and low N applications both in Ox2 and O8 lines (**Figures 6**, **7**). However, the Ox2 was significantly higher than WT in early stage and late stage (28 days) in high nitrogen application, but there was no difference after 14 days of transplanting (**Figures 6B, D**). In case of O8 the *pmoA* microbial population was increased as compared to WT after 28 days in both low and high N application (**Figures 7B–D**).

The *nirK* abundance of WT at the mid-tiller stage was significantly higher in Ox2 and O8 lines than WT in both low and high N regimes (**Figures 8**, **9A, D**). The trend of *nirS* microbial abundance changing with time was not consistent in Ox2 and O8 even at different N regimes (**Figures 8**, **9B, E**). The *nosZ* abundance in the Ox2 line was significantly higher than that of WT in the early stage of rice after transplanting, showed an opposite trend in the middle stage, and no significant difference in the later stage in both low and high N application (**Figures 8C, F**). A similar trend in line O8 compared to its WT was observed (**Figures 9C, F**).

### Paddy soil from cultivars regulated by nutrient use efficiency

Paddy soil is a complex ecological environment. Many factors influence physical and chemical properties in paddy soil. The Ox2 and O8 lines didn’t show any impact on soil pH compared to their WT (**Table 1**). The available P (AP) was significantly lower in O8 than its WT during low N application. But no significant difference was recorded for AP in both overexpress lines in low or high N (**Table 1**). The available K (AK) was significantly higher in both overexpress lines (Ox2, O8) even in low and high N regimes except the O8 line in high N application was not significant than WT (**Table 1**).

**Table 1:** The chemical characteristics i.e., pH, available phosphorus (AP) mg/kg, available potassium (AK) mg/kg, Total Nitrogen (TN) g/kg, Total organic carbon (TOC) g/kg, ammonium NH4+-N mg/kg and nitrate N0_3_N in mg/kg in low and high nitrogen application from the rhizosphere soil of the WT, Ox2, WT-N and O8 in the field experiment.

Genotype	pH	AP(mg/kg)	AK(mg/kg)	TN(g/kg)	TOC(g/kg)	NH^+^_4_-N (mg/kg)	NO^-^_3_ (mg/kg)
WT (low nitrogen)	6.52±0.10a	48.82±6.02ab	111.00±29.88b	0.57±0.24a	17.49±1.13a	4.53±0.24a	1.08±0.06a
Ox2(low nitrogen)	6.56±0.06a	43.35±5.10b	157.00±20.26a	0.68±0.12a	16.74±0.17ab	4.15±0.09a	1.09±0.06a
WT-N (low nitrogen)	7.21±0.02a	37.50±2.45a	104.23±9.91b	0.59±0.23b	21.26±1.12b	4.25±0.25b	0.87±0.0ca
O8 (low nitrogen)	7.24±0.02a	32.15±1.26ab	123.58±16.59a	0.62±0.25a	22.24±1.29ab	4.46±0.26a	1.10±0.10b
WT (high nitrogen)	6.60±0.03a	39.86±3.39a	99.50±13.18b	0.63±0.06a	16.85±0.28b	4.94±0.70b	1.14±0.11a
Ox2 (high nitrogen)	6.62±0.05a	41.99±5.60a	125.50±2.08a	0.55±0.06b	17.73±0.26ab	5.12±0.27b	1.03±0.03b
WT-N (high nitrogen)	7.08±0.08a	34.83±1.88a	88.32±5.32b	0.59±0.24a	24.78±1.77a	4.16±0.25b	1.11±0.09b
O8 (high nitrogen)	7.06±0.10a	31.57±2.69b	87.12±1.96	0.52±0.18b	21.80±1.37b	5.64±0.28a	2.91±0.71a

Significant difference between two lines (transgenic and respective WT) are indicated by different letters: a= high value, b= lower value, *p* values shown with every value where n = 3 (*t*-test).

The total nitrogen content (TN) in rhizosphere soil in WT was significantly higher than Ox2 (**Table 1**). In terms of Ammonium $\left(\text{N}{\text{H}}_{4}^{+}-\text{N}\right)$ and nitrate $\left(\text{N}{\text{O}}_{3}^{-}\text{N}\right)$ in rhizosphere soil, WT and Ox2 showed no significant difference. We recorded similar results in O8 lines in both low and high N regimes. We calculated the above-ground N concentration of Ox2 and O8 under low and high N applications. Under low and high N treatment, the nitrogen concentration of the culm and leaf of WT was significantly higher than Ox2 (**Figure S1A, B**). It was higher in leaf in O8 lines as compared to WT (**Figure S1C**). But when high N was applied, the concentration of N in culm and leaf was lower in O8 than WT-N (**Figure S1D**). However, the nitrogen concentration in the panicle was significantly higher in Ox2 and O8 than in their respective WT, irrespective of high or low N regimes (**Figure S1**). It demonstrates that these transgenic lines used N for high yield production. Therefore total N concentration of Ox2 (**Figures S2A, C**) and O8 (**Figures S2B, D**) in both low and high application of N fertilizer was higher in panicles than their leaf and culm. In addition, grain yield (**Figures S2A-S3A**), grain number per panicle (**Figures S2B**, **S3B**), grain weight (**Figures S2C**, **S3C**), effective tiller (**Figures S2D**, **S3D**), seed setting rate (**Figures S2E**, **S3E**), plant height (**Figures S2F**, **S3F**) and dry biomass (**Figures S4A, B**) were significantly higher in both Ox2 and O8 lines as compared to their WT in both low and high N regimes.

## Discussion

### The co-relation of N use efficient transgenic lines and GHG’s emission

It has been reported that nitrogen-efficient materials can efficiently absorb and transport available N in soil (Chen et al., 2017). Applications of non-sulfate, NH_4_ based (e.g., urea, (NH_4_)_2_HPO_4_) fertilizers have been linked to higher CH_4_ emissions in paddy fields (Kumaraswamy et al., 2000). This is due to the increased plant growth and carbon supply that results from high N applications, which in turn provides more methanogenic substrate and improves the efficiency of CH_4_ transport to the atmosphere (Bodelier et al., 2000; Schimel, 2000; Dong et al., 2011; Abalos et al., 2014). N_2_O originates from both nitrification and denitrification (produced more N_2_O) in paddy. The addition of N fertilizers to the soil directly increases the potential for N_2_O emission (Dobbie and Smith, 2003; Yan et al., 2008). However, N efficient transgenic lines discharge less N_2_O (**Figure 2**) as they utilized accessible N in the rhizosphere, leaving less available N which is vital for N_2_O discharge. Moreover, the long-term N application also regulates the N_2_O discharge in soil. The combined application of organic N and mineral N (inorganic fertilizer) could also mitigate N_2_O emission (Liu et al., 2017; Liu et al., 2020; Yuan et al., 2022).

Our transgenic lines (Ox2, O8) previously reported as high-affinity nitrate transporters cultivars utilize available N in their panicles and grains (**Figures S1**–**S4**). It seems they provide less substrate for CH_4_ emission in paddy soil. The plant growth, grain yield and biomass were significantly increased in Ox2 and O8 lines compared to WT in both high and low N applications (**Figures S2**–**S4**). The high agronomic yield, plant biomass and nitrogen utilization for seeds formation in our results (**Figures S2**–**S4**) are compatible with previous results (Fan et al., 2016; Chen et al., 2017). Here, our cultivars utilized available N and mitigated CH_4_ and N_2_O in soil alternatively (**Figures 1**, **2**). Nitrification and denitrification are linked to N_2_O production in soil. It is an intermediate product of these processes which are influenced by the amount of ammonium and nitrate in the soil and other factors including soil water-filled pore spaces. (Dobbie and Smith, 2003). Soil type and land-use might have direct relationship with nitrification and denitrification to N_2_O production from agricultural soil (Liu R. et al., 2016). Also, after microbial decomposition of N in soil and fertilizers, and N_2_O is often increased where N availability surpasses plant requirements like in paddy conditions. However, in case of our transgenic lines, they didn’t have much available N for nitrification and denitrification than WT in the respective rhizosphere (**Table 1**).

### Relationship of excessive CO_2_ with reduced CH_4_and N_2_O emissions

Most of CH_4_ emissions occur right after transplanting seedlings from nursery into field until first growing stage (Wu et al., 2019). Our results were compatible with previous findings of high discharge in CH_4_ in WT (Iqbal et al., 2021) during an early growth stage except for transgenic lines which show a decline in CH_4_ emission (**Figure 1**). Our results also verified a decrease in N_2_O emission rate (**Figure 2**). Reduced aerenchyma also reduced other gases which affect oxidation of CH_4_ and nitrification of N_2_O (Dobbie and Smith, 2003; Gutierrez et al., 2014).

However, our results revealed higher CO_2_ emission from transgenic (Ox2, O8) lines (**Figure 3**). The relationship of CO_2_ and CH_4_ emission is co-related. Elevated CO_2_ in paddy conditions reduced N_2_O and CH_4_ emission during field conditions (Yu et al., 2022). The reduction in CH_4_ and N_2_O induced by excessive CO_2_ emission in our transgenics is might be attributed to higher soil Eh, higher O_2_ transport into soil. As, CO_2_ and N are potentially involved in production of more tillers and rice biomass (Fan et al., 2016; Yu et al., 2022). However, in some previous findings the elevated CO_2_ also stimulate CH_4_ and N_2_O emission along with high yield in some breeding cultivars (Liu et al., 2018; Hu et al., 2020).

Another potential reason behind high values of CO_2_ and N_2_O from transgenics are different phenotypes as Ox2 is Wuyunjing7 and O8 is Nipponbare background. Different genotype and phenotypic backgrounds possibly produce different amount of yield, plant growth, O_2_ and water usage (Fan et al., 2016; Chen et al., 2017). Therefore, GHG discharge is also associated with said factors (**Figures 2**, **3**).

### Restricted aerenchyma formation regulates GHG emission

Aerenchyma is a specific structure formed by plants to adapt anoxic or anaerobic environment. Here, a primary purpose is to transport oxygen and also a meaningful way to excrete CH_4_ from paddy fields (Sorrell et al., 2013). Limited aerenchyma was one of the additional factors in reducing N_2_O and CH_4_ emissions. It is reported that about 90% of CH_4_ from paddy was released into atmosphere was through aerenchyma, that signifies their role in discharge of CH_4_ and other GHG (Kim et al., 2018; Iqbal et al., 2021). Aerenchyma formation has a regulatory correlation with gaseous exchange in rice. During submerged conditions, aerenchyma provides passage for O_2_ to a tip of the root, then into rhizosphere and removes gases (ethylene, N_2_O, CH_4_ and CO_2_) into soil (Butterbach-Bahl et al., 1997; Colmer, 2003; Colmer and Voesenek, 2009). The continuous formation of aerenchyma in wet plants is inevitable because it provides the passage for gaseous exchange between aerial and anaerobic (flooded) soil (Vartapetian and Jackson, 1997).

Limited aerenchyma in our transgenic lines (**Figures 4**, **5**) also played a significant role in reducing GHG emissions (**Figures 1**, **2**). Limited aerenchyma formation was a crucial factor for less CH_4_ emission in our cultivars. CH_4_ oxidation in plants depends on the transport pathways like aerenchyma formation (Van den Berg et al., 2016). The formation of gaseous spaces during flooded conditions is common in plants. Such plants can directly regulate CH_4_ oxidation. (Wagatsuma et al., 1990). In waterlogged plants (rice, reeds) the GHG emission is controlled by constant existence of aerenchyma in the roots (Wagatsuma et al., 1990; Vartapetian and Jackson, 1997; Van den Berg et al., 2016).

### Methanotrophs impact on GHG emission

Methane production requires the participation of *mcrA* and an anaerobic environment, so there are higher levels of methanogenic bacteria in paddy environment. The methane-oxidizing bacteria will oxidize a portion of methane during methane emission. Therefore, the distribution of methane-oxidizing bacteria increases in soil with high permeability (Judd, 2011). Methanotrophs play a significant role in mitigating GHG. It is reported that some methanotrophs consume 10-30% CH_4_ before it reaches the atmosphere. These bacteria utilize CH_4_ as their sole carbon and energy source (Bodelier et al., 2000; Shrestha et al., 2010). According to an estimation, about 62% of global N_2_O emissions are through natural and agricultural soils, mainly due to bacterial nitrification and denitrification (ammonia oxidation) (Okereke, 1993; Zumft, 1997; Thomson et al., 2012). The information of bacterial populations (methanotrophs, nitrification and denitrification) in soil is directly associated with N_2_O and CH_4_ fluxes from paddies. The CH_4_ flux was positively correlated with *mcrA* and *nosZ* genes and negatively correlated with *pmoA*. Nitrous oxide flux was positively correlated with *pmoA* and *nirK* and negatively correlated with *nosZ* gene abundance (**Figures 6**-**9**). By observing quantitative results of *pmoA*, *nirK* and *nosZ*, it was found that abundance of *pmoA* in Ox2 was higher than WT at some points (**Figures 6**–**9**). The CH_4_ oxidation to ammonia, methanol and hydroxylamine is regulated by mono-oxygenase enzymes, which have homologous copper membrane (Klotz and Stein, 2008). The stimulation or inhibition of methane oxidizing bacteria in rhizosphere depends on N from NO_3_ and NH_4_, carbon from CH_4_ and genetic potential of methane oxidizing bacteria (Stein and Klotz, 2011).

In this study two N efficient transgenic lines were utilized (Ox2, O8) in order to increase N uptake and utilization efficiency, which in turn aimed to increase rice yield and growth. The transgenic cultivars were found to have an impact on the microbes related to CH_4_ and N_2_O emissions (**Figures 6**–**9**). As seen in the abundance of *nosZ* and the cumulative emissions of CH_4_ and N_2_O (**Figures 1E**, **2E**). Additionally, the abundance of certain microbial enzymes such as *pmoA, mcrA* and *nirK* were observed to be affected by the N regimes and growth stages. We found that under paddy conditions, the *nirK* abundance of WT was significantly higher than Ox2 (**Figure 8**). Besides methane and oxygen, N can also have an essential function in CH_4_ oxidation and N_2_O emission and may become an inhibiting or stimulating factor for growth of methanotrophs and nitrifying bacteria (Dobbie and Smith, 2003; Bodelier and Laanbroek, 2004; Smith et al., 2008; Shrestha et al., 2010; Thomson et al., 2012).

The abundance of the microbial enzymes *pmoA* and *mcrA* were observed too be significantly higher at certain growth stages, specifically at 14 days. This was found to correspond with a higher cumulative nitrous oxide emission in WT compared to Ox2 (**Figure 2E**). A similar trend was also observed in the microbial abundance of WT and Ox2 under various other treatments. However, the cultivar O8 did not display a consistent pattern. Additionally, our study found that both high and low N applications played a significant role in the microbial growth, as seen in **Figures 6**–**9**. Microorganisms that use ammonia as an energy source (nitrifiers) and microorganisms that use methane as an energy source (methanotrophs) have many similarities in terms of their energy requirements and enzymes they use, such as methane monooxygenase enzyme, ammonia monooxygenase/particulate enzyme family (Stein et al., 2012). This suggests that the populations of both nitrifying and methanotrophs microorganisms maybe regulated by transgenic cultivars Ox2 and O8 (**Figures 6**–**9**).

## Conclusion

In conclusion, our study demonstrated that the use of Overexpressed transgenic cultivars (Ox2, O8) in paddy fields can effectively regulate yield, nitrogen consumption, oxygen transport and greenhouse gases (CH_4_, N_2_O, CO_2_) emission. These transgenic lines accomplish this through the utilization of excessive nitrogen and carbon dioxide in the rhizosphere, regulation of associated functional microorganisms in the soil, and development of aerenchyma in rice roots.

Our findings provide valuable suggestions and future recommendations:

1. Our findings provide useful insight into the potential for developing sustainable agriculture cultivars that can enhance crop production for food security while also reducing greenhouse gas emissions and addressing the challenge of global warming.
2. Our data highlights the crucial role that nitrogen plays in the emissions of CH_4_ and N_2_O, and suggest that transgenic cultivars (Ox2, O8) that increase N uptake may provide reduced substrate for these emissions.
3. Our study revealed a significant negative correlation between N remobilization into grains and the emission of N_2_O

## Data availability statement

The original contributions presented in the study are included in the article/**Supplementary Material**. Further inquiries can be directed to the corresponding authors.

## Author contributions

MI and YZ considered the experiments and wrote the manuscript. YW, PK, KC and LZ performed the experiments. XF and XX designed, supervised the project, performed the statistical data analysis and wrote the manuscript. All authors contributed to the article and approved the submitted version.
