# Supplementary figures and images for: High-yielding nitrate transporter cultivars also mitigate methane and nitrous oxide emissions in paddy
Source: Front Plant Sci. 2023 Feb 22;14:1133643. doi: 10.3389/fpls.2023.1133643 (PMC9992815; doi:10.3389/fpls.2023.1133643)

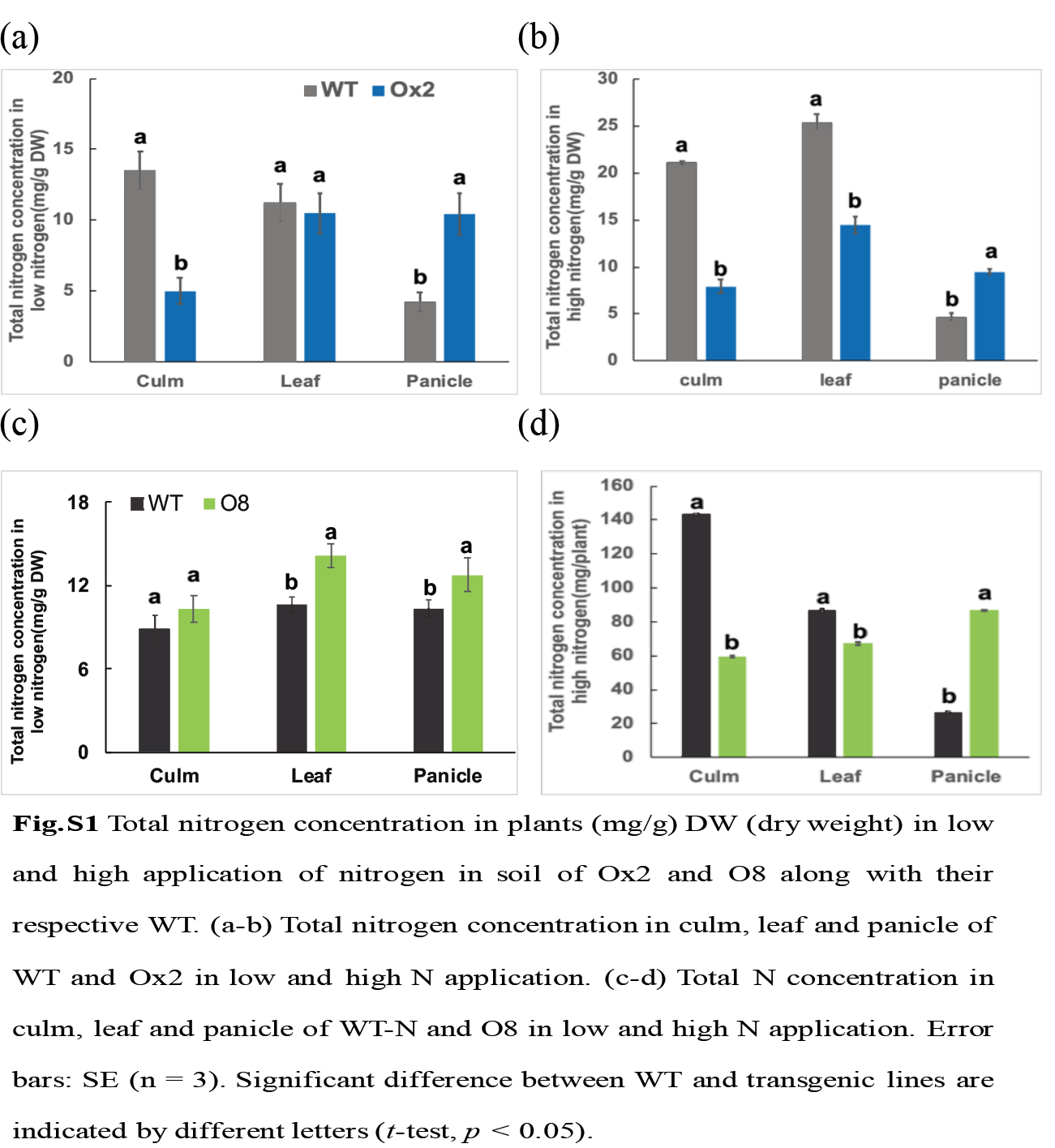

Supplement: Supplementary file 1 [file Image_1.png]

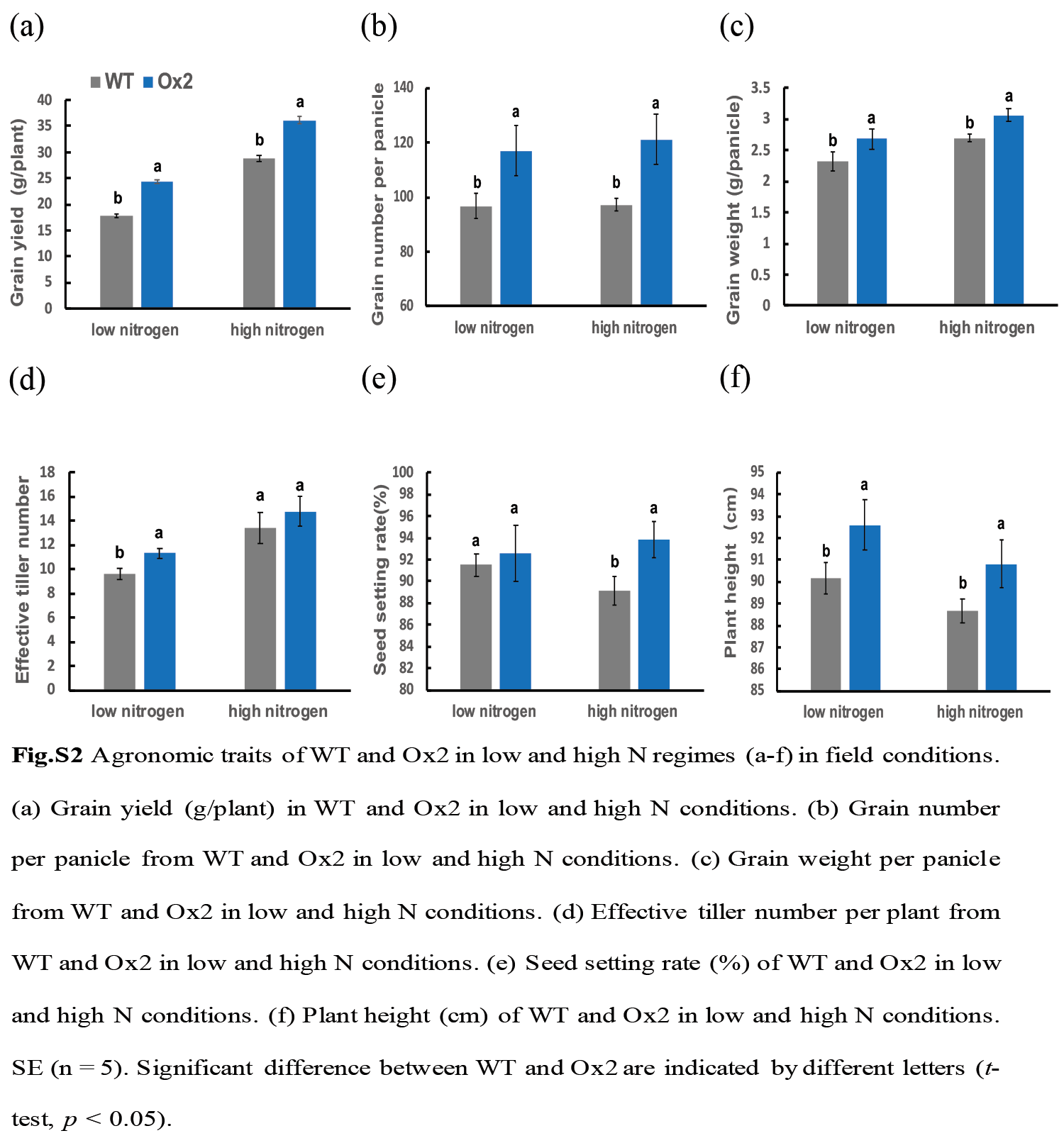

Supplement: Supplementary file 2 [file Image_2.png]

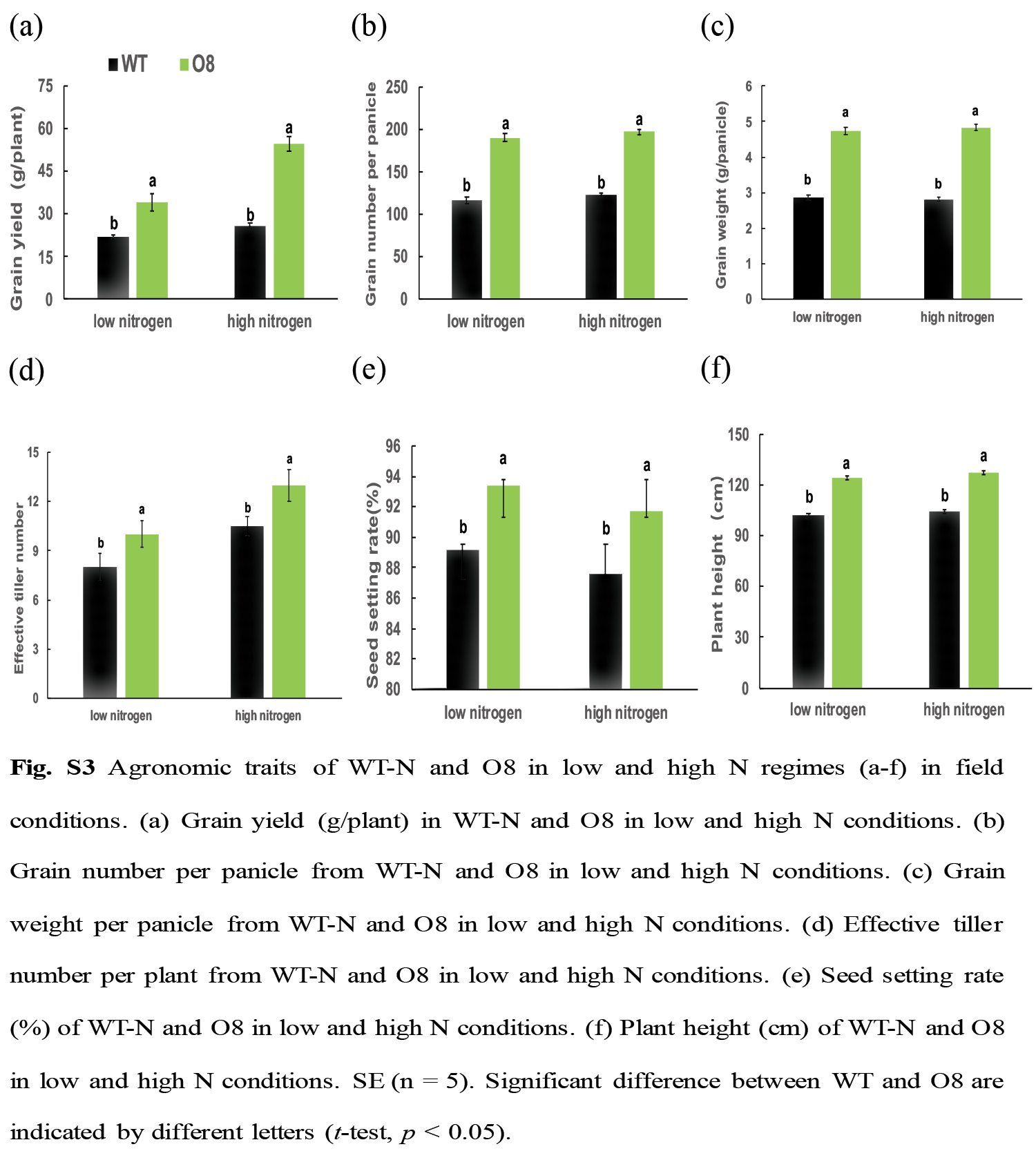

Supplement: Supplementary file 3 [file Image_3.png]

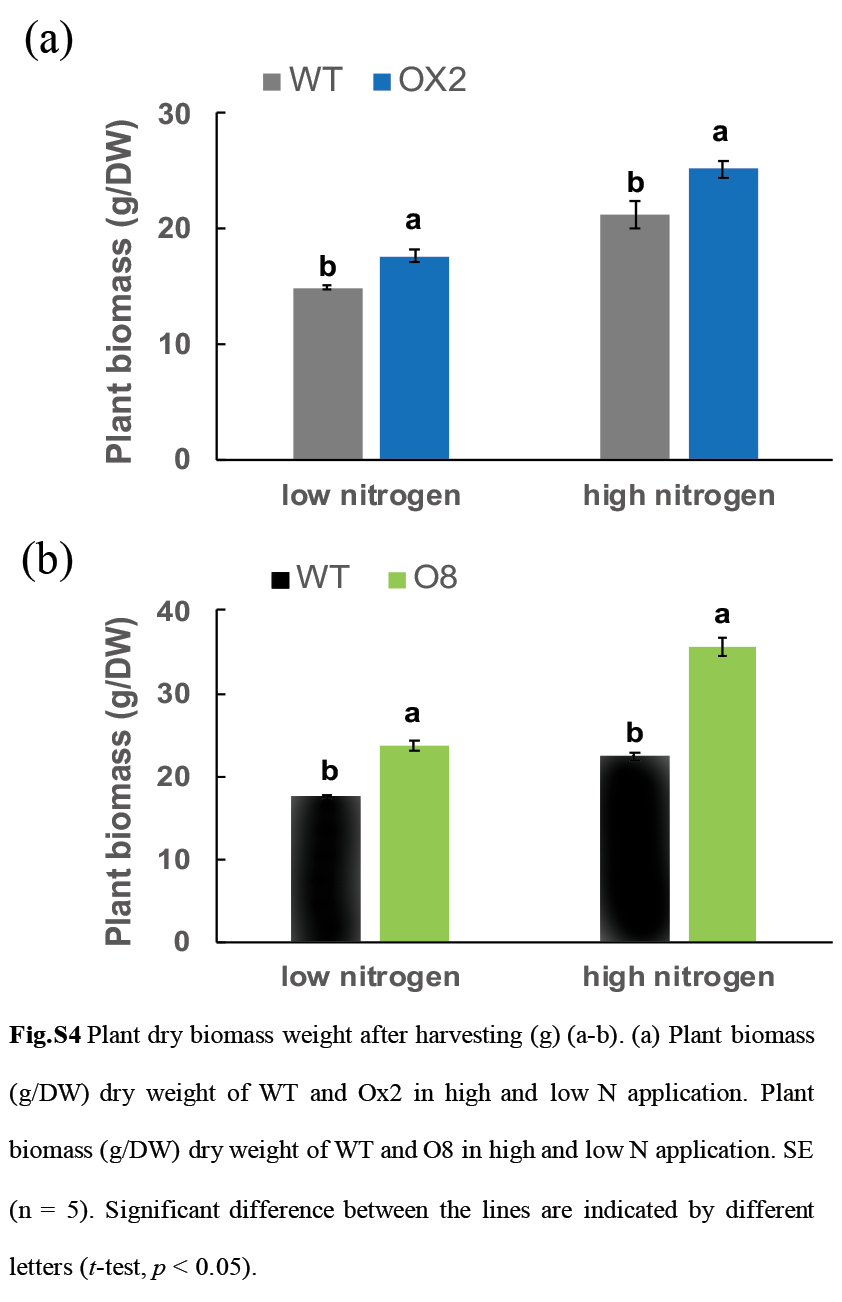

Supplement: Supplementary file 4 [file Image_4.png]
